# Summer Olympic sports and female athletes: comparison of anti-doping collections and prohibited substances detected in Australia and New Zealand vs. France

**DOI:** 10.3389/fspor.2023.1213735

**Published:** 2023-09-08

**Authors:** Corinne Buisson, Lance Brooker, Catrin Goebel, Ryan Morrow, Rima Chakrabarty, Naomi Speers, Adeline Molina, Magnus Ericsson, Katia Collomp

**Affiliations:** ^1^ LADF, French Anti-Doping Laboratory, Université Paris-Saclay, Orsay, France; ^2^ ASDTL, Australian Sports Drug Testing Laboratory, National Measurement Institute, Sydney, NSW, Australia; ^3^ DFSNZ, Drug Free Sport New Zealand, Auckland, New Zealand; ^4^ SIA, Sport Integrity Australia, Fyshwick, ACT, Australia; ^5^ AFLD, French Anti-Doping Agency, Paris, France; ^6^ CIAMS, Université D'Orléans, Orléans, France; ^7^ CIAMS, Université Paris-Saclay, Orsay, France; ^8^ SAPRéM, Université d'Orleans, Orléans, France

**Keywords:** female athletes, summer Olympic sport, Australia, New Zealand, France, anti-doping collection, prohibited substance

## Abstract

Like any athlete, female athletes may be tempted to use prohibited substances during competition or training to enhance their performance. Anti-doping tests performed on female athletes in summer Olympic sports from two geographical areas: Australia/ New Zealand, and France were compared. First, the distribution of sample collections across different sports disciplines, as well as the distribution of substances was investigated. Then the distribution of collections and substances detected in the five sports categories (Strength/Speed, Endurance, Mixed, Motor Skills with High Energy Expenditure, and Motor Skills with Low Energy Expenditure) were studied with consideration of therapeutic use exemptions obtained by the athlete. Australia/New Zealand and France were similar in their overall number of anti-doping collections performed. Likewise, both regions had the same sports disciplines (athletics, aquatics, cycling) and sport categories (Mixed and Endurance) as having the highest number of sample collections. The Motor Skills with High Energy Expenditure, and Motor Skills with Low Energy Expenditure categories had the lowest number of sample collections. However, the number of substances detected was significantly different (*p* < 0.05) with a greater number of substances found in the French data. There were a few substances in common between the two geographical areas, namely prednisone/prednisolone, carboxy-THC, terbutaline, vilanterol and methylphenidate, but most were different. In-competition tests were the category where most of the AAFs were found.

## 1. Introduction

The World Anti-Doping Code International Standard Prohibited List (1) is reviewed annually in consultation with scientific, medical, and anti-doping experts to ensure it reflects current medical and scientific evidence and doping practices. The Prohibited List includes any substance or method that satisfies at least two of the following three criteria: (1) It has the potential to enhance or enhances sport performance; (2) It represents an actual or potential health risk to the Athlete; (3) It violates the spirit of sport (this definition is outlined in the WADC). Some substances and methods are prohibited at all times i.e., In- and Out-of-Competition (IC and OOC) as defined in the Code. This is the case for the substance classes S0 to S5 (non-approved substances, anabolic agents, peptide hormones, beta-2 agonists, hormone and metabolic modulators and diuretics), as well as for the prohibited methods M1 to M3 (manipulation of blood and blood components, chemical and physical manipulation, and gene and cell doping). Other substance classes are only prohibited IC, such as substance classes S6 to S9 (stimulants, narcotics, cannabinoids, and glucocorticoids), and class P1 (beta-blockers) are only prohibited in particular sports (1).

A recent study has shown that women use fewer doping substances than men, with different uses of the substances administered (2). This appears notably as a gendered difference in the use of substance classes, with relatively lower use of hormone modulators and cannabinoids and higher use of beta-2 agonists, diuretics, and glucocorticoids by women. The main limitations with that study were: first that only about 20% of doping tests were conducted on women, limiting the number of samples; second that this study was carried out only in one country (France) and therefore had a limited perspective on the global trends; and last that there was a potential overestimation of positive cases as therapeutic use exemption (TUE) data were omitted. The TUE data would reflect cases where the athlete was permitted to use the prohibited medication or method to ensure that she could compete in a proper state of health.

Therefore, to complement the results of the first study, data for female athletes from the Australia/New Zealand region have been added in this study. This is to compare different regions with respect to the number of samples collected from female athletes in summer Olympic sports, and the prohibited substances and methods detected prior to and after TUEs were granted. This differentiates the use of substances for therapeutic purposes from other purposes (e.g., doping, substance abuse and inadvertent use) by female athletes. We also compare the implementation of anti-doping collections and substance use with reference to both sport disciplines and categories. This is because sport disciplines and categories appear to directly affect the choice of substances used (3).

## 2. Methods

### 2.1. Database

The data used for this study were based on results obtained for doping control tests collected between 2013 and 2022 in the category of summer Olympic sports from three National Anti-Doping Organizations (NADOs): the French Anti-Doping Agency, Sport Integrity Australia, and Drug Free Sport New Zealand. Data from Australia and New Zealand NADOs (AUS/NZ) were pooled due to the similarity of anti-doping practices in these countries and to ensure a comparable number of samples against the French NADO (FR) dataset.

The samples has been analysed by World Anti-Doping Agency (WADA) accredited laboratories in accordance with the WADC International Standard for Laboratories (4). The results were uploaded into the WADA Anti-Doping Administration & Management System (ADAMS) platform.

The anonymized data were extracted from ADAMS with the NADO's consent in April 2023. For each test result, the following information was collected: discipline, gender, test result [negative, adverse analytical findings (AAF, test results confirmed a prohibited substance was present or prohibited method was used) or atypical findings (ATF, test results were not possible to conclude as negative or AAF)], substance detected, substance class, sample type (urine or non-athlete biological passport blood), and year of collection. Data was extracted for female athletes at all levels of competition in the relevant sports. In parallel, anonymized data regarding TUEs obtained by female athletes were reported. Due to this anonymization, it was not possible to know if an athlete was repeatedly tested on different occasions.

Only one substance was reported when the drug and its metabolites were found in the same sample. The number of sample collections and substances detected were reported for each summer Olympic sport (5).

In addition, as there is a sport-specific use of doping substances (3), these sport disciplines were distributed into the following categories: (1) Strength/Speed (ST&SP); (2) Endurance (END); (3) Mixed (MIX); (4) Motor skills with high energy expenditure (MS&HE); (5) Motor skills with low energy expenditure (MS&LE). Tests in swimming, cycling and athletics when no race distance was specified were included in MIX.

### 2.2. Statistics

Standard descriptive statistics were performed in the R programming environment (version 4.1.1) and a Pearson's Chi-square test was used for quantitative comparisons of: (i) the number of anti-doping collections performed in the different sports categories, (ii) the number, and type of class and substances in summer Olympic sports before and after TUE, (iii) the substances used based on the sports categories before and after TUE, with *p*-values ≤ 0.05 considered significant. The Pearson's Chi-square test was chosen as the statistical analysis based on literature on biostatistics (6) and study where quantitative comparisons were performed (7).

## 3. Results

### 3.1. Number of anti-doping collections performed in the different summer Olympic sports disciplines

Over the period 2013–2022, a similar number of blood and urine samples were collected by the two regions (10799 for AUS/NZ vs. 12932 for FR, see Table 1). For both geographic areas, the sports in which the highest number of sample collections were obtained were 1) Athletics, 2) Aquatics, and 3) Cycling.

**Table 1:** Number of anti-doping samples (urine and blood) analysed for AUS/NZ and FR from 2013 to 2022 in Olympic summer sports and number of detections prior to TUE review.

Sports	AUS/NZ			FR		
	Number of samples	% samples/total tests	Number of substances	Number of samples	% samples/total tests	Number of substances
**Aquatics**	**1160**	**10** **.** **7**	**2**	**957**	**7** **.** **4**	**10**
Archery	51	0.5	0	88	0.7	0
**Athletics**	**1481**	**13** **.** **7**	**6**	**3794**	**29** **.** **4**	**28**
Badminton	75	0.7	0	83	0.6	1
Basketball	522	4.8	1	636	4.9	2
Boxing	164	1.5	0	145	1.1	5
Canoe/Kayak	688	6.4	2	254	2.0	0
Climbing	3	0.0	0	48	0.4	0
**Cycling**	**1703**	**15** **.** **8**	**3**	**1164**	**9** **.** **0**	**9**
Equestrian	51	0.5	0	40	0.3	0
Fencing	38	0.4	0	144	1.1	2
Field Hockey	439	4.1	3	36	0.3	1
Football	841	7.8	2	646	5.0	1
Golf	30	0.3	1	48	0.4	1
Gymnastics	200	1.9	0	282	2.2	1
Handball	0	0.0	0	850	6.6	5
Judo	119	1.1	1	427	3.3	0
Karate	7	0.1	0	44	0.3	0
Modern Pentathlon	31	0.3	0	78	0.6	0
Rowing	740	6.9	0	305	2.4	3
Rugby	776	7.2	1	259	2.0	2
Sailing	67	0.6	0	83	0.6	0
Shooting	95	0.9	0	41	0.3	3
Tennis Table	40	0.4	0	76	0.6	0
Taekwondo	58	0.5	0	103	0.8	0
Tennis	42	0.4	0	282	2.2	1
Triathlon	604	5.6	2	904	7.0	13
Volleyball	109	1.0	0	380	2.9	1
Weightlifting	588	5.4	6	574	4.5	5
Wrestling	77	0.7	0	161	1.2	1
TOTAL	**10799**		30*	**12932**		**95**

Data in bold correspond to the sports for which the greatest number of samples was observed.

* *p < 0.05.*

The list of prohibited substances detected in these samples was presented in Table 2. Among the thirty summer Olympic sports studied, only seven presented no prohibited substances (Archery, Climbing, Equestrian, Karate, Modern Pentathlon, Sailing, Taekwondo, Table Tennis) wherever anti-doping collections occurred.

**Table 2:** Prohibited substances detected in the samples collected by AUS/NZ and FR over the period 2013–2022. Substances in bold and underlined correspond to TUEs approved by testing authorities.

Sports	S1 (AUS/NZ)	S1 (FR)	S2 (AUS/NZ)	S2 (FR)	S3 (AUS/NZ)	S3 (FR)	S4 (AUS/NZ)	S4 (FR)	S5 (AUS/NZ)	S5 (FR)	S6 (AUS/NZ)	S6 (FR)	S7 (AUS/NZ)	S7 (FR)	S8 (AUS/NZ)	S8 (FR)	S9 (AUS/NZ)	S9 (FR)	P1 (AUS/NZ)	P1 (FR)
Aquatics	1 Ligandrol					2 terbutaline		1 GW1516			1 sibutramine	1 tuaminoheptane						2 **budesonide**		
																		4 **prednisone/olone**		
Archery																				
Athletics	1 Norethandrolone	1 tibolone 3 19-Na 1 boldenone 1 trenbolone 1 clenbuterol		5 EPO	1 higenamine	1 salmeterol		1 trimethazidine		1 furosemide	1 methylphenidate 1 phenethylamine	2 heptaminol						1 **budesonide** 8 **prednisone/olone** 2 **triamcinolone** 1 fluticazone		
	1 Testosterone																			
Badminton																		**1 prednisone/olone**		
Basket	1 Zeranol									1 furosemide								1 prednisone/olone		
Boxing								1 tamoxiphene		1 brinzolamide				1 bupremoprhine				1 prednisone/olone		
Canoe /kayak					1 higenamine												**1 prednisone/olone**			
Climbing																				
Cycling				1 EPO						1 chlortalidone	**1 pseudoephedrine** 1 methylphedinate	1 heptaminol			1 Carboxy- THC			6 **prednisone/olone**		
Equestrian																				
Fencing										1 indapamide		1 tuaminoheptane								
Field hockey					1 terbutaline						1 methylphenidate	1 amfetamin					1 prednisone			
Football									1 probenicid		**1 modafinil**	1 amfetamin								
Golf	1 Ostarine																			1 propanolol
Gymnastics												1 heptaminol								
Handball						**1 terbutaline**				1 furosemide						2 Carboxy-THC		1 prednisone/olone		
Judo											**1 phentermine**									
Karate																				
Modern Pentathlon																				
Rowing												1 methylphenidate		1 morphine				1 prednisone/olone		
Rugby											1 metamfetamine			1 morphine				**1 prednisone/olone**		
Sailing																				
Shooting										1- hydrochloro-thiazide		1 heptaminol						**1 prednisone/olone**		
Table tennis																				
taekwondo																				
Tennis						**1 vilanterol**														
Triathlon	1 Ostarine				**1 vilanterol**	1 terbutaline						1 heptaminol		2 morphine				2 **budesonide**		
																		7 **prednisone/olone**		
Volleyball																1 Carboxy-THC				
Weightlifting	2 Turinabol										1 methylhexaneamine 1 d-amfetamine							1 fluticasone 1 prednisone/olone		
	1 Ligandrol																			
	1 Ostarine																			
Wrestling												1 methylhexanamine		1 morphine						
												1 heptaminol								
												1 methylphenidate								

### 3.2. Number of anti-doping collections performed in the different sports categories

Sport disciplines from summer Olympic sports were classified into five categories (ST&SP, END, MIX, MS&HE, MS&LE) as described in Table 3. The distribution of the samples collected among these five categories of sports was presented in Table 4. A similar distribution could be observed between the two geographical areas with MIX sports with the highest number of anti-doping tests. For both areas, the second category with the most tests was END, and then ST&SP. Only few sample collections were performed for the category MS&LE (< 2%).

**Table 3:** Distribution of Olympic summer sports and disciplines studied into five sport categories: (1) strength/speed (ST&SP); (2) endurance; (3) mixed (MIX); (4) motor skills with high energy expenditure (MS&HE); (5) motor skills with low energy expenditure (MS&LE).

Strength/Speed (ST&SP)	Endurance	(Mixed) MIX	Motor skills with high energy expenditure (MS&HE)	Motor skills with low energy expenditure (MS&LE)
Aquatics—Swim sprint	Aquatics—Open Water, Swim long distance	Aquatics—Swim middle, Water polo	Aquatics—Artistic swimming, Diving	Archery
Athletics—sprint, jump, throw	Athletics—long distance, marathon, race walk/road, cross country	Athletics—Combined event, Middle distance, tracks	Climbing	Golf
Canoe—sprint	Canoe—long/ocean	Badminton	Cycling—BMX	Shooting
Cycling—sprint	Cycling—road, mountain, cross, track and endurance	Basketball	Equestrian	
	Triathlon	Canoe—middle distance, slalom	Fencing	
		Field hockey	Gymnastics	
		Football	Sailing	
		Handball	Table tennis	
		Rowing	Pentathlon	
		Rugby	Boxing	
		Tennis	Judo	
		Volleyball	Taekwondo	
			Wrestling	
			Karate	

**Table 4:** Distribution of the samples collected by AUS/NZ and FR among the five sport categories (ST&SP, eND, MIX, MS&HE, MS&LE). Distribution of the tests performed IC and OOC.

Sport Category	AUS/NZ	FR	AUS/NZ %	FR %
ST&SP	2107	1774	19.5	13.7
MIX	4954	5525	45.9	42.7
END	2403	3701	22.3	28.6
MS&HE	1159	1755	10.7	13.6
MS&LE	176	177	1.6	1.4
Total	**10799**	**12932**		
IC	3969	7431	36.8	57.5
OOC	6830	5501	63.2	42.5

The distribution between IC and OOC tests was significantly different (*p < 0.05*) between the regions. A lower number of OOC vs. IC tests was observed in FR (42.5% vs. 57.5%) whereas a greater proportion of OOC tests was performed in AUS/NZ (63.2% vs. 36.8%).

### 3.3. Number, and type of class and substances in summer Olympic sports before and after TUE

The distribution by class of prohibited substances found in the anti-doping tests performed by AUS/NZ and FR before and after the removal of AAFs with valid TUEs were presented in Table 5. The proportion of AAFs obtained IC and OOC was also presented there. Most of the AAFs were found IC, with 90% of AAFs in FR found IC and 60% of AAFs in AUS/NZ found IC.

**Table 5:** Distribution of the substances found in anti-doping tests performed by AUS/NZ and FR by class of prohibited substances before and after the removal of AAF with valid TUEs. The proportion of AAF obtained IC and OOC is also presented. For IC testing, the number of substances prohibited at all times (PAT) and prohibited IC only (PIC) are also indicated.

Class	AUS/NZ before TUE	AUS/NZ after TUE	FR before TUE	FR after TUE
S1	11	11	7	7
S2	0	0	6	6
S3	4	3	6	4
S4	0	0	3	3
S5	1	1	7	7
S6	11	8	14	14
S7	0	0	6	6
S8	1	1	3	3
S9	2	1	42	21
P1			1	1
Total	**30**	**25**	**95**	**72**
IC (PAT/PIC)	**20 (9/11)**	**15 (8/7)**	**87 (23/64)**	**65 (22/43)**
OOC	**10**	**10**	**8**	**7**

A significantly higher number of substances (*p < 0.05*) was found in FR samples before and after TUE review. Indeed, compared to AUS/NZ, FR athletes used a higher number of glucocorticoids (FR: 42 vs. AUS/NZ: 2 before TUE; and FR: 41 vs. AUS/NZ: 1 after TUE review). The distribution of substances used by FR athletes covers all the classes (apart S0 = non-approved substances, M1 = manipulation of blood, M2 = chemical and physical manipulation and M3 = gene and cell doping) whereas no substances were found in classes S2 (peptide hormones, growth factors and related substances), S4 (hormone and metabolic modulators) and S7 (narcotics) for AUS/NZ athletes.

Some substances including prednisone/prednisolone, carboxy-THC, terbutaline, vilanterol or methylphenidate were found in both geographical zones, yet in most classes different substances were detected. The substance detected the most times in both geographical areas was prednisone/prednisolone.

The TUEs were observed mainly for substances in the classes S3 (beta-2 agonists), S6 (stimulants), and S9 (glucocorticoids) with a higher number of TUEs associated with AAFs in FR (23 in FR vs. 5 in AUS/NZ) mainly IC (1 substance OOC: terbutaline in FR). Two substances were found to be covered by TUE in both geographical areas: prednisone/prednisolone (S9 glucocorticoids) and vilanterol (S3 beta-2 agonists).

### 3.4. Substances used based on the sports categories before and after TUE

**Figure 1:**
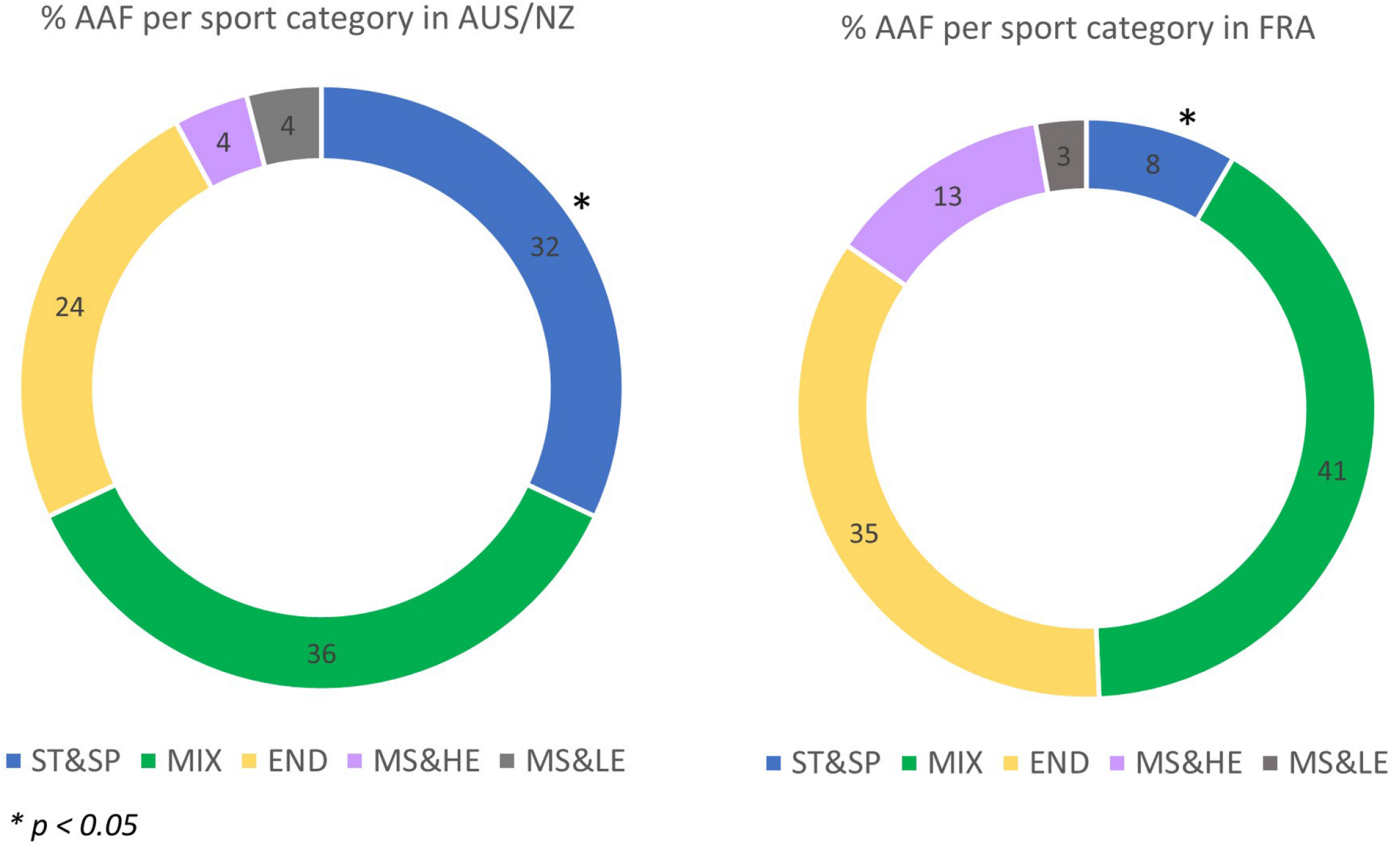
Repartition of AAF after TUE review by sport categories in AUS/NZ and FR.

The distribution of AAFs after TUE review in the sport categories are presented in Figure 1. While the proportion of AAFs are similar in most of the categories, a significant difference (*p < 0.05*) was observed for ST&SP (32% of AAFs for AUS/NZ vs. 8% for FR). The highest percentage of AAFs was observed in MIX sports for both regions which aligns with the larger number of tests performed in this sport category.

**Figure 2:**
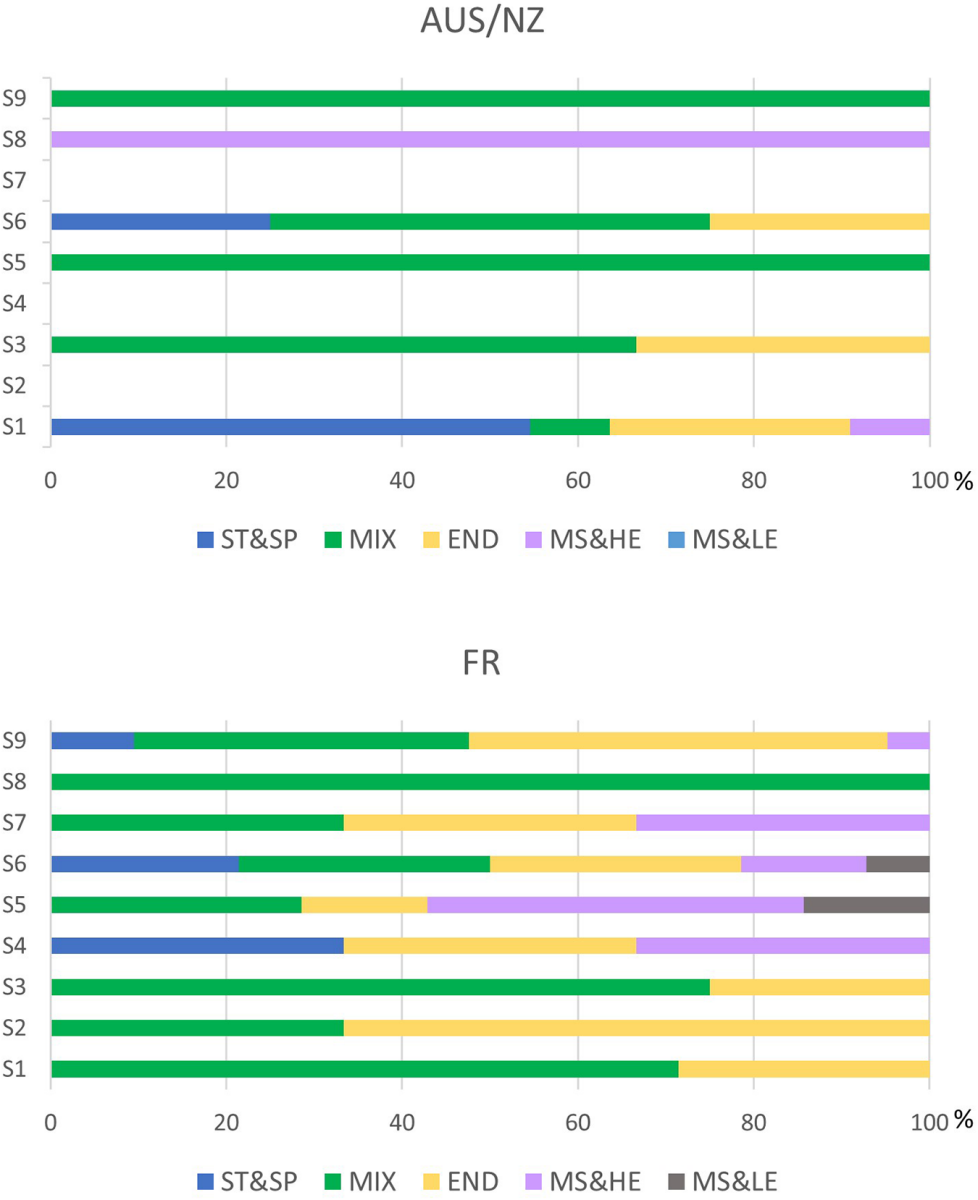
Repartition of AAFs classes among the sport categories after TUE review.

The distribution of AAFs classes among the sport categories after TUE was presented in Figure 2. Different trends were observed between the two geographical areas. While substances from all of the classes apart from S8 (cannabinoids) were found for END sports in FR, only substances from classes S1 (anabolic agents), S3 (beta-2 agonists), and S6 (stimulants) were found in AUS/NZ samples for this category. For MIX sport, similar trends were observed with substances found from most of the categories in which substances have been found. Substances from class S9 (glucocorticoids) were mostly used in END and MIX in FR whereas only in MIX in AUS/NZ. For ST&SP sport, substances from class S1 (anabolic agents) were found only in AUS/NZ whereas substances from class S4 (hormone and metabolic modulators) were found only in FR. Substances from class S6 (stimulants) were detected in ST&SP, MIX and END categories in both FR, and AUS/NZ.

## 4. Discussion

Regarding the number of tests carried out over the decade studied, we obtained a comparable total of anti-doping tests performed between FR and AUS/NZ. This may seem surprising at first glance, as the French population is much larger than that of Australia and New Zealand combined, but these countries compensate with a higher level of sports participation and elite sport results. Indeed, if we look at the number of medals won by female athletes at the last two Summer Olympic Games covered in the present study, we note for the 2016 and 2020 Olympic Games respectively: 13 and 22 medals for AUS, 11 and 11 medals for NZ and 11 and 15 medals for FR female athletes. There is of course a variation in the number of tests according to sport disciplines practiced in these different countries, but the same top 3 for sports the most controlled was found (i.e., athletics, aquatics, and cycling). Additionally, there was a similar distribution between the different categories of sports established, within both FR and AUS/NZ, very few tests were performed in MS&LE.

However, we can note a significantly higher number of IC AAFs in FR when compared to AUS/NZ before and after TUE, with a similar number of detections during OOC testing (both before and after TUE). A hypothesis to partially explain this observation is the difference between the number of substances prohibited IC when compared to OOC. A greater number of substances are included in IC testing, as more substances classes are included, and most tests in FR (57.5%) were IC. In comparison, in AUS/NZ most tests were OOC (63%). Thus, in the FR AAFs after TUE, across the 65 substances detected during IC testing, 43 substances (of which 21 are glucocorticoids) were part of the IC testing classes (60% of the total number found) and 22 substances were part of the OOC testing classes of substances prohibited at all times.

In the AUS/NZ AAFs after TUEs, over the 15 substances detected during IC testing, 7 substances were part of the IC testing classes only and 8 substances were part of the OOC testing classes. It thus appears that regardless of the region, most detections were made during competitions.

Interestingly, we can clearly notice a geographical and/or drug availability impact. Indeed, among all substances detected for stimulants, only 3 (methylphenidate, methylhexanamine, amphetamine) out of 11 were found in both FR and AUS/NZ. For narcotics, while no AAFs were found for AUS/NZ in the studied population, 6 AAFs of which 5 morphine were declared in FR This observation can be explained by the fact that morphine or its pro-drugs such a codeine are frequently used in France (7, 8). Similarly, the higher number of glucocorticoids found in FR samples may reflect their very common oral prescription in France for many pathologies including various anti-inflammatory diseases or asthma (9), and better awareness for physicians providing prescriptions for athletes and of athletes regarding TUE requests seems warranted.

A limitation of this study is that WADA-accredited laboratories may ask the testing authorities if a TUE exists before conducting a confirmation procedure on certain categories of substances for which there is a presumptive adverse analytical finding [PAAF, suspicious results after the initial testing procedure (ISL, WADA)]. This can significantly decrease the number of AAF for substance classes such as beta-2 agonists, stimulants, or glucocorticoids where TUEs were pre-approved, and the testing authorities gave their approval not to conduct confirmation procedure.

Research into female athletes is lacking generally. Indeed, there are very few studies on the effects of prohibited substances and methods on female athletes, but by extrapolating the research regarding male athletes, the use of peptide hormones, growth factors, related substances and mimetics and glucocorticoids classes in MIX and END categories is consistent with the literature. Indeed, the substances in both classes (mainly EPO and prednisone/prednisolone in the present study) have been shown to significantly improve only aerobic performance (10–13). Similarly, it seems rational to find the use of stimulants in ST&SP, MIX and END categories, as acute stimulant administration has demonstrated ergogenic effects on aerobic and anaerobic performances (14, 15). In both AUS/NZ and FR samples, beta-agonists were found in MIX and END categories where their acute and short-term effects on performance were assessed (16–18). In the AUS/NZ samples, there was a classical predominance of the use of anabolic agents in ST&SP sports categories, but anabolic agents were primarily found in the MIX sport categories in the FR samples, where strength and power may also play a key role (19). Regarding the use of diuretics, most were found in MS&HE sports, which include a large part of the disciplines with weight categories. Finally, in view of the small number of substances found in classes of hormone and metabolic modulators, narcotics, cannabinoids, and beta-blockers, it is not possible to interpret their use.

It is interesting to analyse the distribution of the different classes presumed used for doping purpose according to the type of sport categories, considering that unintentional use cannot be excluded. Indeed, while a similar proportion was found for the MIX categories (i.e., 36% and 41% in AUS/NZ and FR samples respectively), we found a greater representation of prohibited substances in ST&SP sports in AUS/NZ compared to FR samples (AUS/NZ: 32%; FR: 8%) when expressed as a percentage of the total number of substances. The difference between countries disappears when expressed in the percentage of the number of samples collected (AUS/NZ: 0.38%; FR: 0.34%). However, a greater proportion was found in FR vs. AUS/NZ END samples (i.e., 35 vs. 24%), the difference being maintained when expressed in the percentage of the number of samples collected (respectively 0.68% and 1.03%).

## 5. Transparency statement

The present article untitled “summer Olympic sports and Female Athletes: Comparison of anti-doping collections and prohibited substances detected in Australia and New Zealand vs. France” does not contain any identifiable samples. All data is a fully anonymized anti-doping data. All data which could identify an individual athlete was removed before provision by the relevant organizations. Under the requirements of the World Anti-Doping Code, Laboratories are blind to the identification of the athlete (20).

At the provision of samples, athletes do provide consent for their de-identified data to be used for research purposes. An example of this consent is: “*Your sample as well as data derived from your Personal Information may also be used for secondary purposes such as anti-doping research or to improve and verify the quality of anti-doping detection methods if the conditions of Code Article 6.3 are met, namely: measures are adopted to ensure your Personal Information and sample cannot be linked to each other and cannot be traced back to you; the research or quality improvement study complies with applicable law and internationally recognized ethical research principles*”.

The data were obtained from the WADA's Anti-Doping Administration & Management System (ADAMS) database with the consent from the National Anti-Doping Organizations involved in this article.

## 6. Conclusion

This study detected similarities and differences between FR and AUS/NZ. Over the period 2013 to 2022, a comparable number of anti-doping tests was performed with the same top three sports being the most tested. In-competition remains the category of sample collections where most of the AAFs were found. Prednisolone/prednisolone was found to be overall the most detected substance. This study also highlighted the few numbers of sample collections performed for MS&HE and MS&LE sport which makes interpretation of these results difficult.

## Data Availability

The datasets presented in this article are not readily available because of confidential data. Requests to access the datasets should be directed to Corinne Buisson, c.buisson-ladf@universite-paris-saclay.fr.
